# Improvement in physical and mental health attributable to the affordable care act

**DOI:** 10.3389/frhs.2025.1466958

**Published:** 2025-11-26

**Authors:** Jangho Yoon, Beth Hawks

**Affiliations:** 1 Department of Preventive Medicine and Biostatistics, School of Medicine, Uniformed Services University of the Health Sciences, Bethesda, MD, United States; 2 Department of Medicine, School of Medicine, Uniformed Services University of the Health Sciences, Bethesda, MD, United States

**Keywords:** affordable care act, health care reform, health outcomes, difference-in-differences, quantile regression

## Abstract

**Introduction:**

The Affordable Care Act (ACA) represents the most comprehensive U.S. health reform since Medicare and Medicaid. However, evidence on its impact on population health in the general U.S. population, particularly mental health, remains limited.

**Methods:**

We analyzed a nationally representative sample of non-elderly adults aged 18–64 from the Medical Expenditure Panel Survey (2007–2019). Outcomes included two health-related quality of life (HRQOL) measures derived from the SF-12 v2: physical component summary (PCS) and mental component summary (MCS) scores. Using conditional-mean and quantile-regression difference-in-differences models, we examined the effect of the ACA by comparing pre-post changes in PCS and MCS scores among non-elderly adults relative to counterfactuals from TRICARE beneficiaries not subject to ACA provisions.

**Results:**

Our conditional-mean DID estimates indicate that the ACA was associated with a 2.7% increase in PCS scores among non-elderly adults during 2011–2013. Although statistically insignificant, MCS scores exhibited increases of growing magnitude following the implementation of the major ACA major provisions in 2014. Notably, simultaneous-quantile DID estimates suggest that the increases in PCS and MCS scores attributable to the ACA were concentrated among individuals with relatively lower health levels, particularly those around the 30th to 60th percentiles of the score distributions.

**Discussion:**

Findings indicate that the ACA led to measurable gains in physical and mental health, particularly among relatively lower-middle levels of physical and mental health. Policymakers assessing the value of the ACA, or more generally debating the value of expanding access to health insurance in the population, should consider these positive gains in population health.

## 1 Introduction

The Patient Protection and Affordable Care Act (ACA) represents the most comprehensive health care reform implemented in the U.S. since the creation of Medicare and Medicaid in 1965 (1). The ACA includes numerous provisions to achieve its overarching aims: increase access to health care, transform health care delivery and payment systems, improve quality of care, and control health care costs (1–3). Due to the legislation's size and complexity, provisions rolled out systematically following its passage in 2010 (2). For example, as of July 2010, private insurers could no longer deny insurance coverage for children under 19 with preexisting medical conditions (non-exclusion of the preexisting conditions) (2). Young adults aged 18–25 began gaining insurance coverage under their parent's health insurance plan in September 2010 (known as dependent coverage expansion) (4). More significant changes occurred in 2014 when individuals became required to purchase minimum essential health insurance coverage with federal subsidies for low- to middle-income individuals in the Health Insurance Marketplaces, and 25 states (including the District of Columbia) opted to expand Medicaid for low-income individuals (2).

The literature documents a range of benefits of ACA implementation, such as decreases in uninsured individuals (5, 6), improved access to health care services (7–9), and reductions in racial and ethnic disparities in health insurance coverage (7). The individual mandate and ACA Medicaid expansion jointly led to a substantial decrease in the uninsured population, resulting in a 5.9% increase in insurance coverage (10). In particular, Courtemanche and colleagues (9, 11) analyzed post-ACA data on non-elderly adults from the Behavioral Risk Factor Surveillance System and suggested improvement in self-assessed general health status (reporting “very good” or “excellent” overall health) attributable to the major ACA provisions implemented in 2014.

Greater access to health care via ACA implementation may improve health outcomes for newly covered individuals (11). As such, enhanced benefit designs also come with the potential for improved health outcomes. Among other mechanisms, the essential health benefits provision requires that private and public health insurance plans cover comprehensive preventive care, wellness, chronic disease management, immunization, cancer screening, diet counseling, tobacco cessation, birth control, well-woman visits, substance use screening, and depression screening (12–16). Arguably, providing the core benefits package, health services previously often neglected and left out of insurance coverage, can improve the population's health additively and progressively.

This study extends the literature in several crucial ways. First, we assessed population health levels using health-related quality of life (HRQOL) measures validated in the general population and across various health conditions (13–15), separately for physical and mental health. We thereby extended a dichotomized self-assessed general health status measure analyzed in Courtemanche et al. (11, 17). Second, we use quantile regression to explore the heterogeneous effects of the ACA across the full range of health distribution to perform a more comprehensive assessment of its impact. The ACA could improve access to health care, particularly among those who need health services but cannot acquire health care due to cost. Therefore, improving access to affordable health insurance coverage may result in more pronounced improvements in health status among those individuals in the lower parts of a population's health distribution. The literature is still limited in that prior studies only investigated average conditional mean relationships between ACA implementation and health outcomes. Third, we investigated whether the effect of the ACA accumulated over time. Although some of the ACA's provisions, such as the non-exclusion of preexisting conditions and dependent coverage expansion, became effective with bill passage, other major ACA provisions were phased in over time. Notably, the two most prominent coverage expansion leverages, the individual mandate and state Medicaid expansion, went into effect in 2014 with a potentially higher-dose impact. Also, the effect of the ACA can accumulate over time, and its impact on population health outcomes, in particular, may only be partially visible in the short term. Fourth, our study period spanned both pre- and post-ACA periods, beginning in 2007, allowing us to assess the effect of the ACA as a whole. In contrast, prior studies have largely focused on the post-ACA era with an emphasis on Medicaid expansion (18), or on specific provisions, such as dependent coverage expansion (19) and protection for individuals with pre-existing conditions (20). Fifth, we leveraged data on TRICARE beneficiaries to construct valid counterfactuals in outcomes trends against which we compared outcome trends in the non-elderly adult population affected by the ACA. TRICARE, the U.S. Department of Defense's health care program, provides universal coverage to ∼9.6 military beneficiaries (21) and is exempt from ACA provisions. TRICARE beneficiaries included in our analysis comprised dependents of uniformed service members as well as military retirees and their dependents. This population was not directly influenced by ACA implementation as TRICARE was not subject to ACA provisions.

## 2 Materials and methods

### 2.1 Data source and sample

We pooled annual data from the Medical Expenditure Panel Survey (MEPS) Household Components (HC) for 2007 through 2019. The MEPS is a nationally representative health care survey of the U.S. civilian noninstitutionalized population. The MEPS HC fields questionnaires to individual household members to collect detailed data on health care utilization and cost, health insurance coverage, and health status. The MEPS employs a complex survey design that features stratified and multi-stage sampling and the oversampling of certain groups, such as minorities and low-income families until 2015 (22). For example, those surveyed for MEPS go through five rounds of interviews over two years, and a subset of these sampled households are selected to participate in the next year's survey (22).

The sample for this study included all non-elderly adults aged 18–64 in the MEPS (*n* = 262,045), including civilian TRICARE beneficiaries (*n* = 4,907) and non-TRICARE civilians (*n* = 257,138). The Institutional Review Board at [blinded for review] approved the research.

### 2.2 Measures

We examined two continuous health outcome measures: Physical Component Summary (PCS) and Mental Component Summary (MCS) scores derived from the Short-Form 12 Version 2 (SF-12v2) (23, 24). The SF-12v2 is a shorter alternative to the SF-36 Health Survey (25) and contains 12 items capturing physical functioning, role limitations, pain, general health, vitality, social functioning, and mental health. PCS and MCS scores were calculated using the standard SF-12v2 scoring algorithm and adjusted to U.S. population norms (mean = 50, SD = 10). Higher scores reflect better physical or mental health, respectively. The summary scores have been validated in the general population and various conditions (13–15).

The main explanatory variables included indicators of the policy intervention group and post-ACA period(s). The policy intervention group variable equals 1 for non-elderly adults not enrolled in TRICARE and, therefore, subject to ACA implementation. We assigned 0 for TRICARE beneficiaries who were exempt from ACA implementation. The *overall* post-ACA period variable indicates observations for the years 2011 through 2019. Major ACA coverage expansion provisions, such as the individual mandate and state Medicaid expansions, went into full effect in January 2014. Thus, we also augmented the analysis by splitting the post-ACA period into the *short-term* (years 2011–2013) and *long-term* (years 2014–2019) post-ACA periods.

### 2.3 Analyses

We utilized difference-in-differences (DID) analysis to compare pre-post changes in the health outcome measures for the policy intervention group of non-TRICARE civilian individuals with counterfactual pre-post changes for the comparison group of TRICARE counterparts. Our identification strategy capitalizes on the fact that TRICARE provides universal health coverage and access to care for all beneficiaries during the pre- and post-ACA period and is not subject to ACA implementation.

In our benchmark conditional-mean DID regression model specification, we modeled each health measure as a function of the interaction term of the policy intervention group and post-ACA period indicators. The coefficient on the interaction term captures a conditional-mean incremental change in the outcomes attributable to ACA implementation under the conventional “parallel trend” assumption, discussed below.

All regression models included the policy group indicator (to capture baseline heterogeneity between the policy intervention and comparison groups), the post-ACA period indicator (to adjust for an average pre-post change in the outcomes due to unobserved factors), and quadratic time trends (to capture unmeasured factors that explain overall trends in the measures during the study period). We also controlled for demographic and socio-economic characteristics that are likely to influence health status, including age, sex, race/ethnicity [Black, all other race, and Hispanic (reference: non-Hispanic White)], marital status [widowed, divorced, separated, and single (reference: married)], census region of residence [northeast, mid-west, and west (reference: south)], education [high school graduate, college graduate, and graduate school (reference: <high school)], and family income [near poor, low-income, middle-income, high-income (reference: poor)].

We adopted a generalized linear modeling (GLM) procedure to estimate the empirical DID models and exploited the identity link function and gamma distribution family. Estimates were survey-weighted, and standard errors were adjusted for clustered and stratified sampling of MEPS data.

To examine the ACA's short-term vs. long-term effects, we augmented the conditional-mean DID model by specifying the short-term and long-term post-ACA period indicators and their interaction terms with the policy intervention group indicator.

We augmented the conditional-mean DID models with simultaneous-quantile DID regression (26, 27) and analyzed the heterogeneous effects of the ACA on health by the level of health. This extension allowed us to examine the outcomes being studied more comprehensively, at different percentiles of the distributions of PCS and MCS scores, not just at the conditional means of the distributions. Standard errors came from 200 bootstrap repetitions. Supplementary Appendix A includes additional details on our analytic approaches.

The DID approach yields unbiased estimates under the parallel trend assumption that the policy intervention and comparison group's outcome measures evolved in parallel before the ACA. First, we do not know of any changes to the TRICARE policy that would have led to divergent trends in the health measures pre-ACA. Visually, aggregate yearly time-series data on the health measures overall exhibited that both policy intervention and comparison groups had similar pre-ACA trends (Supplementary Appendix B). We also performed several falsification regression analyses (Supplementary Appendix C). We checked whether there was a difference in time trends between the policy and comparison groups using the pre-ACA subsample (i.e., 2007–2010). We did this by examining the coefficient on the interaction term of the time trends and policy group indicator and the effect of placebo policies by using leads of ACA implementation. All falsification tests supported the validity of our empirical approach.

## 3 Results

In the pre-ACA period (2007–2010), non-elderly adults under the policy intervention and TRICARE beneficiaries exhibited both systematic differences and comparability across sociodemographic and health-related characteristics (Table 1). Compared with the policy intervention group, TRICARE beneficiaries were older, more likely to be married, less likely to be Hispanic but more likely to be Black, more concentrated in the South, and reported higher levels of education and family income, along with higher unemployment rates. Despite these differences, several other dimensions suggest comparability between the two populations. Both groups were predominantly White, had nearly identical gender distributions, similar divorce/separation rates, substantial representation in the West, high school completion as the modal educational attainment, and comparable proportions of families in the middle-income range (200%–400% FPL). Taken together, while the policy intervention and comparison groups differed across several observable characteristics, they also shared meaningful similarities.

**Table 1:** Demographic characteristics of the sample, 2007–2019 (unweighted).

Characteristics of the sample	Non-elderly adults			Tricare beneficiaries		
	Pre-ACA, 2007–2010 (%)	Post-ACA, 2011–2013 (%)	Post-ACA, 2014–2019 (%)	Pre-ACA, 2007–2010 (%)	Post-ACA, 2011–2013 (%)	Post-ACA, 2014–2019 (%)
Race/ethnicity						
Non-Hispanic White (reference)	71.2	68.1	70.0	67.5	61.4	65.3
Black	18.6	20.5	17.9	21.9	26.7	23.9
Other Race	8.9	10.1	10.1	9.6	10.1	9.2
Hispanic	27.1	30.6	29.1	12.7	15.7	14.0
Age in years [# (S.D.)]	39.7 (13.2)	39.6 (13.4)	40.5 (13.5)	43.5 (14.3)	43.8 (14.1)	43.5 (14.2)
Female	53.0	52.9	52.9	52.4	47.8	47.4
Marital status						
Married (reference)	51.7	47.4	47.0	69.8	66.7	67.6
Widow	1.8	1.7	1.8	2.4	4.7	3.1
Divorced	11.0	10.8	11.2	9.1	10.5	9.3
Separated	3.0	3.4	3.0	3.6	3.0	2.6
Single	32.5	36.8	37.0	15.0	15.2	17.4
Census region						
South	37.3	37.4	37.4	58.6	52.0	56.2
Northeast	15.3	16.4	15.8	6.1	7.3	6.7
Midwest	20.1	18.6	19.7	9.6	14.0	11.7
West	27.3	27.6	27.1	25.7	26.7	25.4
Education						
<high school	26.8	23.7	21.7	11.6	8.4	7.8
High school	44.1	37.7	40.0	49.0	42.7	39.8
College	22.3	31.4	29.5	32.0	39.4	39.8
>college	6.8	4.3	7.3	7.4	5.8	10.7
Unemployed	28.5	31.5	28.0	36.3	38.1	36.5
Family income						
Poor: <100% FPL	17.6	20.0	17.5	8.5	10.0	8.0
Near poor: 100% to <125% FPL	5.5	6.1	5.2	3.3	3.5	3.8
Low: 125% to <200% FPL	15.8	17.0	15.2	12.0	13.3	11.3
Middle: 200% to <400% FPL	30.8	29.5	29.6	33.7	34.9	29.6
High: ≥400% FPL	30.2	27.4	32.5	42.5	38.4	47.4
Health measures						
PCS score [# (S.D.)]	50.7 (9.7)	50.6 (9.7)	51.0 (9.4)	48.8 (11.1)	47.8 (11.5)	49.0 (10.6)
MCS score [# (S.D.)]	50.4 (10.1)	50.9 (10.1)	51.7 (9.5)	51.0 (10.2)	51.1 (10.0)	51.3 (10.0)
*n*	78,618	65,851	112,669	1,370	1,173	2,364

Concerning physical health status, the policy intervention group had a slightly higher mean PCS score than TRICARE beneficiaries before ACA implementation (50.7 vs. 48.8), and the pattern was persistent post-ACA. PCS scores, in general, dropped for both population groups (and more substantively for TRICARE beneficiaries) in the short-term post-ACA period (2011–2013). In the long-term post-ACA period (2014–2019), PCS scores, on average recovered to levels slightly higher than the pre-ACA scores for both groups (51.0 vs. 49.0). MCS scores were higher for TRICARE beneficiaries only slightly before ACA implementation (50.4 vs. 51.0) but increased at a higher rate among non-TRICARE individuals. Between the pre-ACA and long-term post-ACA periods, the average MCS score increased by 2.6% from 50.4 to 51.7 for the policy intervention group, compared to a 0.6% increase from 51.0 to 51.3 for TRICARE beneficiaries.

**Figure 1:**
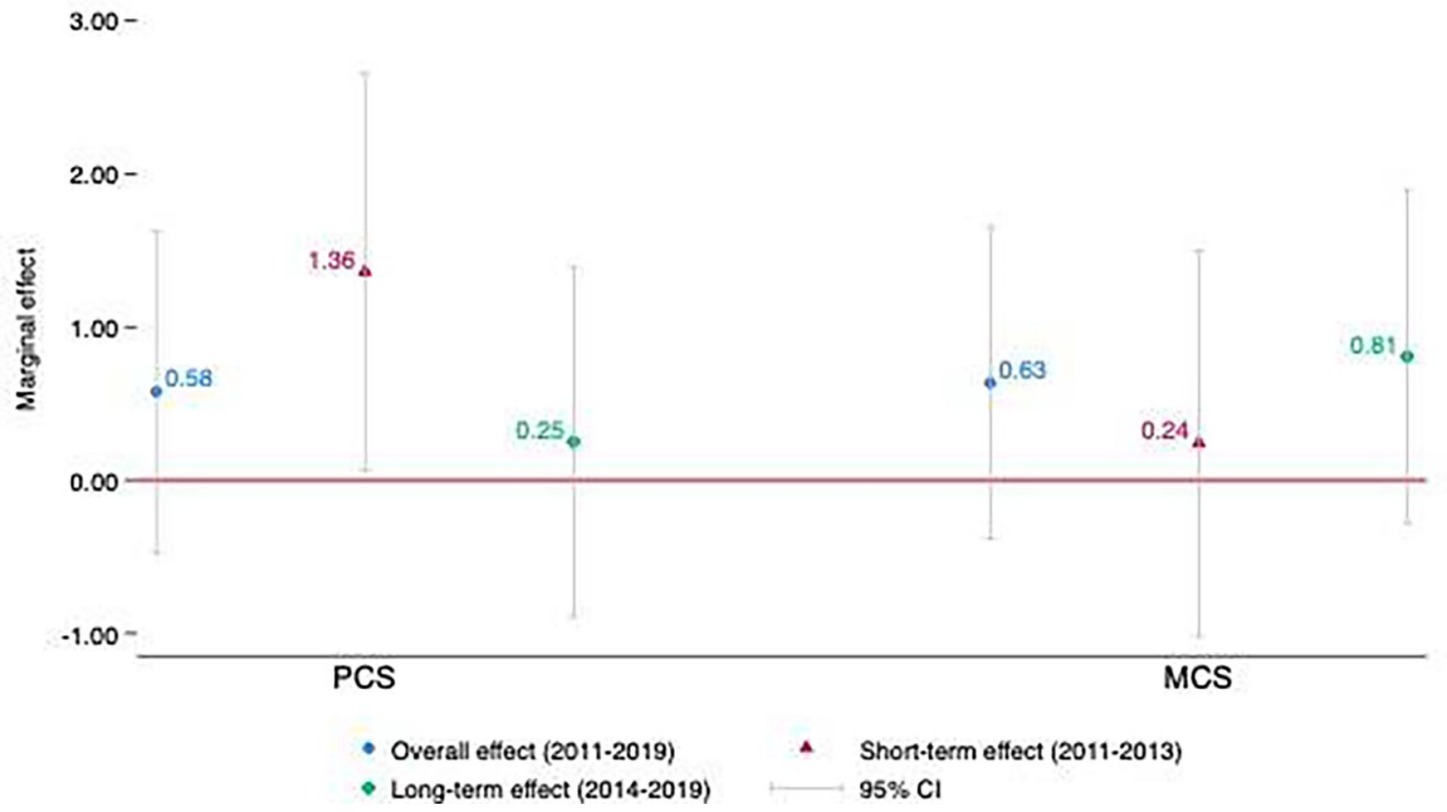
Average treatment effects of ACA (for non-TRICARE sample) on PCS and MCS scores, 2011–19.

Adjusted DID estimates of the conditional-mean effects of the ACA on PCS and MCS scores are presented in Figure 1, separately for the overall (2011–2019), short-term (2011–2013), and long-term (2014–2019) post-ACA periods. The estimated effects of the ACA were always positive although generally not statistically discernable. Notably, the ACA was significantly associated with an increase in PCS score by 1.36 points (*p* = 0.04) over the short-term post-ACA period of 2011–2013. This change equates to a 2.7% increase from the pre-ACA mean PCS score for the policy intervention group. MCS scores show a gradual upward pattern following ACA implementation, with DID estimates increasing from 0.24 points in 2011–2013 to 0.81 points in 2014–2019. Although these estimates do not meet conventional thresholds for statistical significance, the observed trend implies a potentially meaningful improvement in population mental health when considered from the perspective of practical significance. Full regression results provided in Supplementary Appendix D.

**Figure 2:**
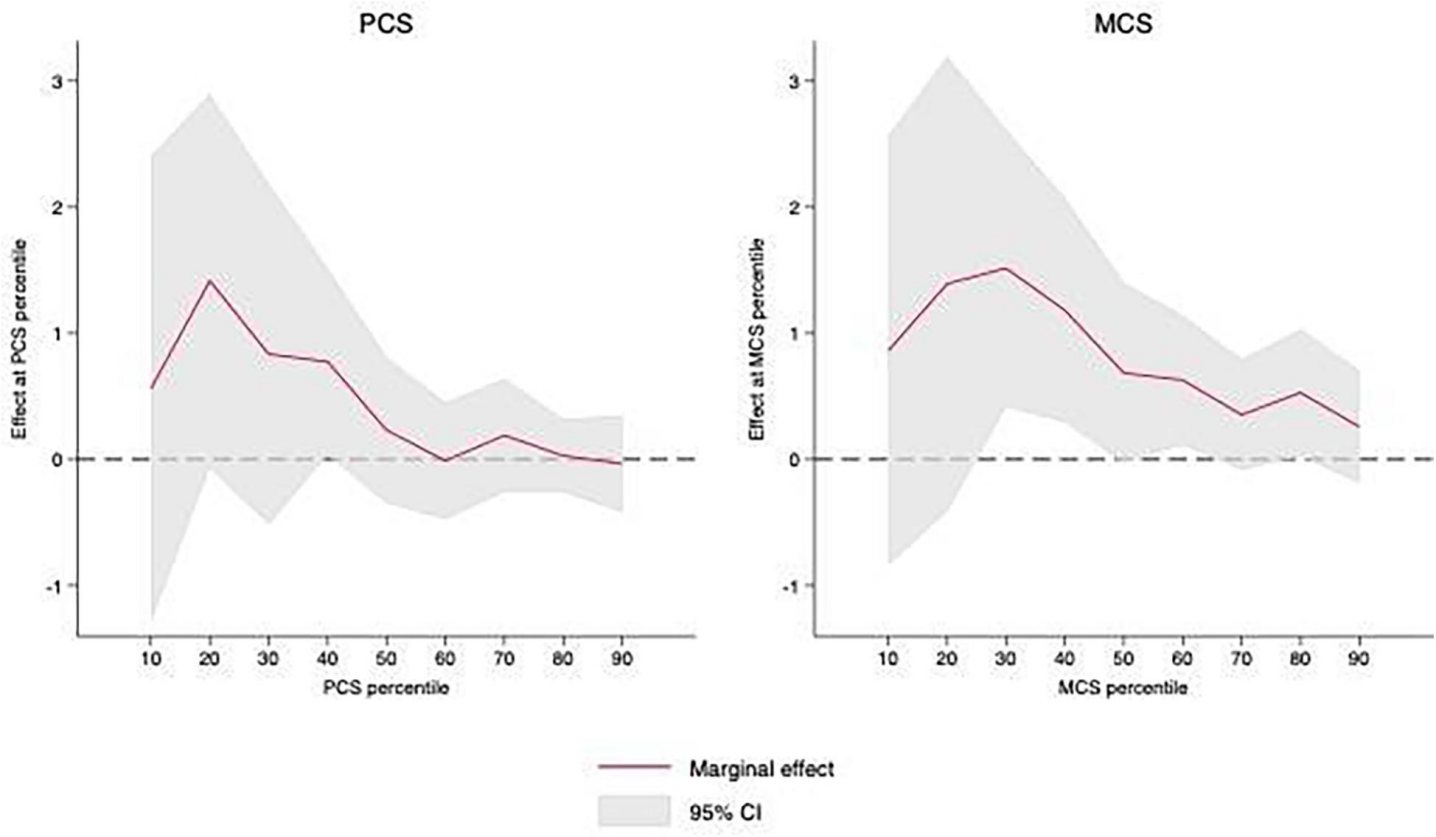
Average treatment effects of ACA (for non-TRICARE sample) across percentiles of PCS and MCS scores, 2011–19.

Figure 2 illustrates the estimated percentile-specific effects of the ACA across the entire ranges of PCS and MCS scores for the post-ACA period (2011–2019). The ACA was statistically significantly associated with an increase in PCS scores around the 40th percentile and MCS scores across the 30th, 40th, and 60th percentiles. Aside from statistical significance, however, the effects of the ACA were generally larger in magnitude and more variable at lower PCS and MCS scores. Taken together, the findings may suggest a beneficial effect of the ACA on physical and, in particular, mental health for individuals with relatively mid to lower health levels. See Supplementary Appendix E for full results.

**Figure 3:**
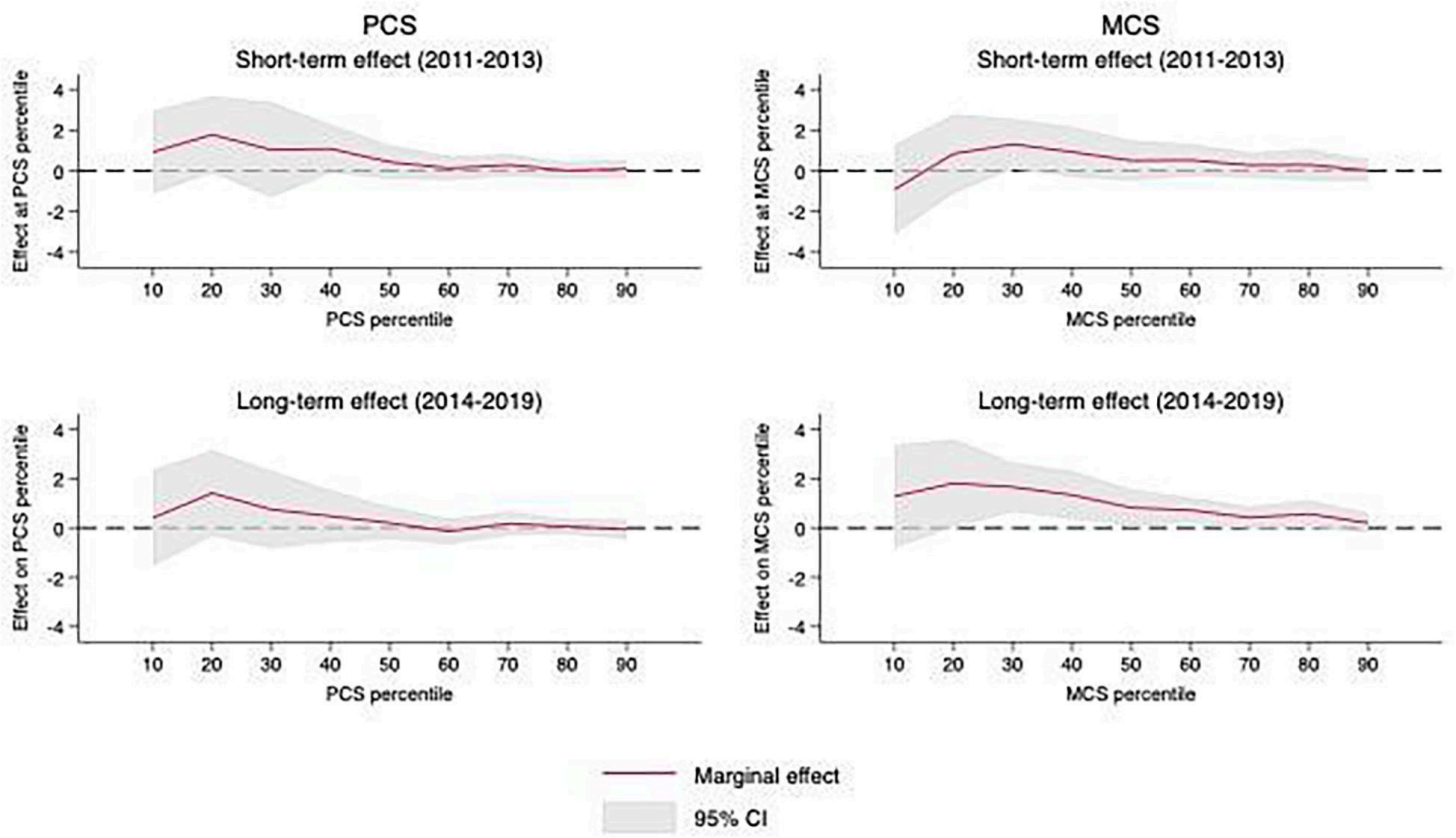
Average short- and long-term treatment effects of ACA (for non-TRICARE sample) across percentiles of PCS and MCS scores, 2011–19.

Figure 3 depicts positive short- and long-term effects of the ACA across the full distributions of PCS and MCS scores. The estimated relationships were generally larger in magnitude and more variable at lower levels of PCS and MCS scores in both the short and long terms. Nevertheless, these associations only reached statistical significance at the 95% level for PCS scores at the 20th percentile during the 2014–2019 post-ACA period. In comparison, the effect of the ACA on MCS scores overall grew over time, increasing in both magnitude and statistical significance in the long term. The ACA was statistically significantly associated with increases in MCS scores across the lower-to-middle segments of the MCS score distribution (30th, 40th, and 60th percentiles) during the 2014–2019 post-ACA period. Detailed results appear in Supplementary Appendix F.

### 3.1 Supplemental analyses

We performed several supplemental analyses to assess the robustness of our main findings (Supplementary Appendix G). We checked the sensitivity of our main DID estimates to baseline differences in observable characteristics between the policy intervention and control groups. Following Stuart et al. (46), we re-estimated the regression models using inverse-probability-weights (IPW) to adjust for between-group and pre-post compositional differences and thereby minimize selection-on-observable bias in pooled cross-section DID analysis (Supplementary Appendix H). The between-group and pre-post compositional differences in the observable characteristics either disappeared or substantially reduced after weighting. Importantly, the IPW-DID estimates are comparable to the main estimates.

We compared our primary intent-to-treat (ITT) framework with a treatment-on-the-treated (TOT) approach. The ITT approach captures the population-level effect of the ACA by including both compliers and non-compliers, regardless of health insurance status, thereby incorporating impacts through all possible pathways including potential spillover and indirect impacts. By contrast, the TOT approach re-estimates the models excluding the uninsured, thereby isolating the effect of the ACA among compliers (i.e., individuals who obtained coverage). Results from the TOT analysis were consistent with the main ITT findings, suggesting that the observed effects are not the artifact of the analytic framework.

The raw aggregate pre-ACA trends in the outcomes (Supplementary Appendix B) did not appear perfectly parallel. To address this concern, we followed the approach of Bilinski and Hatfield (28), allowing for group-specific overall time trends. We also permitted differences in overall time trends between the pre-and post-ACA periods. We identified and excluded influential observations using the difference-in-fits statistics and a cutoff value suggested by Belsley et al. (29). Across these robustness checks, our main conclusions remained consistent, especially for the lower-to-middle segments of the PCS and MCS score distributions. All analyses were conducted using Stata 18 MP (StataCorp LP, College Station, TX).

## 4 Discussion

This study examined the impacts of the ACA on population physical and mental health, as measured by validated HRQOL measures. Our analysis highlights the importance of federal health reform in improving the population's health, suggesting potential gains in both physical and mental health attributable to ACA implementation. Specifically, the physical health score increased by 2.7% in the early phase of ACA implementation (2011–2013) relative to its pre-ACA level among non-elderly adults. This overall finding qualitatively aligns with Courtemanche et al. (11) which analyzed data from the 2011–2018 Behavioral Risk Factor Surveillance System and reported improved self-assessed “very good” or “excellent” health status due to ACA implementation. We also found progressive gains in population mental health as the ACA's major provisions came into force in 2014, although these improvements did not reach statistical significance.

Importantly, this study is the first to report that the beneficial effects of the ACA on population health were more conspicuous among individuals with relatively lower health levels. This result may imply that the ACA benefits those targeted by the federal health reform, specifically individuals at a greater risk of ill health and with limited access to health care services. Given that such individuals are more frequently found in lower socio-economic strata, our finding might translate into reduced health inequity in the population, prompting further investigation. Further, we discovered increased variability in the ACA's health impacts among individuals at lower physical and mental health levels. A potential explanation is that the ACA could have led to more heterogeneous responses to improved access to care among those with lower health levels because relatively more important roles of social determinants of health (SDOH) for this population, such as family income, poverty, access to healthy foods, quality housing, and transportation to medical appointments. Future studies are needed to better understand the mechanisms that underly this finding.

As the most significant health insurance expansion in recent decades, the ACA has brought the uninsured rate to its lowest recorded level (30). Subsequent policies such as the American Rescue Plan Act (ARPA) and the Inflation Reduction Act (IRA) further sustained the reduced uninsurance rate through enhanced Marketplace premium subsidies (31, 32). The current policy direction, however, highlights a departure from this trajectory. Enhanced premium subsides are scheduled to sunset at the end of 2025 absent legislative action, and efforts to reinstate the short-term limited duration insurance (STLDI)—plans exempt from ACA regulations (33), offering lower premium but less comprehensive coverage—are being advanced. In addition, federal policy increasingly promotes greater state autonomy through Medicaid work requirements and potential block grants or per capita caps for Medicaid financing (34). While these approaches are framed as promoting consumer choice and market-based solutions, our study underscores that the ACA's coverage expansion was associated with potential improvements in health outcomes, particularly among less healthy individuals, suggesting that rolling back these gains could undermine important public health benefits.

Given that the federal health reform that emphasized expanding health insurance coverage may result in improved health, as our study demonstrates, it is essential to understand a value-based health insurance design that may maximize population health gains. For example, the mandatory “essential health benefits” covered under the ACA include, among others, comprehensive preventive care services and chronic disease management (such as immunization, cancer screening, diet counseling for those at higher risk of chronic conditions, tobacco cessation, birth control, and well-woman visits, substance use screening, and depression screening). The essential health benefits requirement might elicit better population health outcomes even in a relatively short time of its implementation, given that a vast literature linking health insurance coverage to improved access to care (7, 8, 10, 11, 35–38), better health outcomes and reduced mortality (6, 11, 36, 39). Such a value-based insurance design also acknowledges the crucial role of SDOH in individual and population health. Health plans increasingly invest in a sustainable SDOH infrastructure to address non-medical barriers to health at the patient and community levels (40). Therefore, whether through the ACA or alternative policies that enhance access to affordable, value-based coverage while integrating SDOH, is a promising next step to sustain improvements in population health.

### 4.1 Limitations

There are a few noteworthy limitations of our research. First, we could not isolate the heterogeneous effects of major ACA provisions, such as the individual mandate and state Medicaid expansion. Notwithstanding, Courtemanche et al. (9) suggested that compared to other ACA provisions, the effect of Medicaid expansion was marginal in terms of a self-assessed overall health indicator for “very good or excellent” physical health (17). Second, recent advancements in the DID literature (41–43) caution that static DID estimates might be biased under differential treatment timing. For instance, in our study, the timing of Medicaid expansion under ACA varied across states. Following the lead of 24 states and the District of Columbia, which adopted Medicaid expansion under ACA on January 1, 2014 (44), 16 additional states extended their Medicaid programs in May 2023 (45). Therefore, the effects of ACA may be staggered due to multiple intervention timing of Medicaid expansion by states. Unfortunately, the datasets used in this study did not allow us to exploit such state variation, and our results could overstate the actual effect of ACA under the presence of positive, beneficial effects of ACA. Third, the relatively smaller sample size of TRICARE beneficiaries compared with the policy intervention group of non-TRICARE adults may limit statistical power and precision in estimating treatment effects, although the DID framework with survey weights helps address these imbalances. Fourth, we limited our analyses to 2019 because the COVID-19 pandemic could obscure the true relationships. Because ACA's health impacts may further accumulate beyond 2019, future studies should expand the follow-up period and address the intricated effects of the pandemic to assess the full implications of ACA on health. Lastly, the ACA might have had a spillover effect on TRICARE beneficiaries, especially for those receiving care outside of the direct military health care system. Although small in number, those enrolled in some TRICARE plans such as TRICARE Reserve Select, TRICARE Retired Reserve, TRICARE Young Adult, and Continued Health Care Benefit Program might choose to purchase coverage through the Health Insurance Market Place with federal premium assistance. We could not explore the potentially positive externality because the MEPS data includes only a few individuals enrolled in those TRICARE plans.

## 5 Conclusion

The ACA led to modest but meaningful improvements in physical and mental health among non-elderly adults. These marginal gains were more pronounced among individuals with relatively lower physical and mental health levels. Specifically, the effect on mental health became pronounced following the ACA's major provisions, which came into effect in 2014.

## Data Availability

We are employed by the US government, but we do not speak for them, which is why they ask that we use the standard language of: The opinions and assertions expressed herein are those of the author(s) and do not reflect the official policy or position of the Uniformed Services University of the Health Sciences or the Department of Defense.

## Data availability statement

Publicly available datasets were analyzed in this study. This data can be found here: https://meps.ahrq.gov/data_stats/download_data_files.jsp
