# Supplementary material for: Improvement in physical and mental health attributable to the affordable care act
Source: Front Health Serv. 2025 Nov 26;5:1466958. doi: 10.3389/frhs.2025.1466958 (PMC12689885; doi:10.3389/frhs.2025.1466958)
Supplement: Supplementary file 1 [file Datasheet1.pdf]

Appendix Material

This appendix provides additional details on the methods and analysis described in the main paper.

Appendix A. Econometric models

A1. Conditional-mean difference-in-difference (DID) regression model:

We estimated the following *benchmark* model for each health outcome measure:

(A.1) $h_{igt} = G(\beta_0 + \beta_1 \cdot policy_g + \beta_2 \cdot post_t + \beta_3 \cdot policy_g \times post_t + x'_{igt} \cdot \beta + \tau_t + \tau_t^2) + \varepsilon_{igt}$

Here h_{igt} refers to the PSC or MCS score of individual i of the policy/control group g in year t. G is a function that links the explanatory variables to the outcomes. The "$policy$" variable is the indicator for the policy intervention group of the non-elderly, non-TRICARE adults with the comparison group of non-elderly adult TRICARE beneficiaries. This approach controls for unobserved bias, overall between-group differences in the outcomes that remained after controlling for unobserved individual characteristics. The "$post$" variable indicates the post-ACA implementation period (years 2011-2019) and captures average pre-post changes in the outcomes that were not attributable to the ACA. The interaction term "$policy \times post$" is the variable of primary interest and its coefficient "β_3" measures incremental changes in PCS and MCS scores attributable to the ACA. The x' is a vector of covariates including age, sex, race/ethnicity (Black, all other race, and Hispanic; reference: non-Hispanic White), marital status (widowed, divorced, separated, and single; reference: married), census region of residence (northeast, mid-west, and west; reference: south), education (high school graduate, college graduate, and graduate school; reference: < high school), and family income (near poor, low-income, middle-income, high-income; reference: poor). We also controlled for the quadratic time trend (τ and τ^2).

Generalized linear model (GLM) was used to fit the empirical model specification. For continuous PCS and MCS scores, we used the identity link function and gamma distribution family. Estimates were survey-weighted, and standard errors were adjusted for clustered and stratified sampling of MEPS.

A2. DID regression models for short- vs. long-term effects of ACA:

To examine short- and long-term effects of ACA, we replaced the post-ACA indicator in Eq. (A.1) with a set of two post-period indicators, $post11\_13$ for 2011-2013 and $post14\_19$ for 2014-2019, as well as their interaction terms, $policy \times post11\_13$ and $policy \times post14\_19$, as follows:

(A.2) $h_{igt} = G(\beta_0 + \beta_1 \cdot policy_g + \beta_2 \cdot post11\_13_t + \beta_3 \cdot post14\_19_t + \beta_4 \cdot policy_g \times post11\_13_t + \beta_5 \cdot policy_g \times post14\_19_t + x'_{igt} \cdot \beta + \tau_t + \tau_t^2) + \varepsilon_{igt}$

In this model, the two interaction terms are of main interest. Their coefficients, β_4 and β_5, measure short-term (immediately in 2011-2013) and long-term (later in 2014-2019) changes in PCS and MCS scores attributable to the ACA.

A3. Simultaneous-quantile DID regression models:

We utilized simultaneous quantile regression models to examine heterogeneous effects of ACA on PCS and MCS scores across their entire distributions:

(A.3) $Q_q(h_{igt}) = \beta_0 + \beta_1 \cdot policy_g + \beta_2 \cdot post_t + \beta_3 \cdot policy_g \times post_t + x'_{igt} \cdot \beta + \tau_t + \tau_t^2 + F_{\varepsilon_{igt}}^{-1}(q)$

(A.4) $Q_q(h_{igt}) = \beta_0 + \beta_1 \cdot policy_g + \beta_2 \cdot post11\_13_t + \beta_3 \cdot post14\_19_t + \beta_4 \cdot policy_g \times post11\_13_t + \beta_5 \cdot policy_g \times post14\_19_t + x'_{igt} \cdot \beta + \tau_t + \tau_t^2 + F_{\varepsilon_{igt}}^{-1}(q)$

Here q refers to a quantile of PCS/MCS scores and $Q_q(h_{igt})$ denotes the conditional quantile regression function of PCS/MCS scores at q^{th} (i.e., first quantile, second quantile, etc.) percentile conditional on explanatory variables. $F_{\varepsilon_{igt}}^{-1}$ is the distribution function of the error term ε. We obtained bootstrapped standard errors with 200 repetitions.

Appendix B. Trends in mean PCS and MCS scores for the policy intervention and control groups, 2007-2019

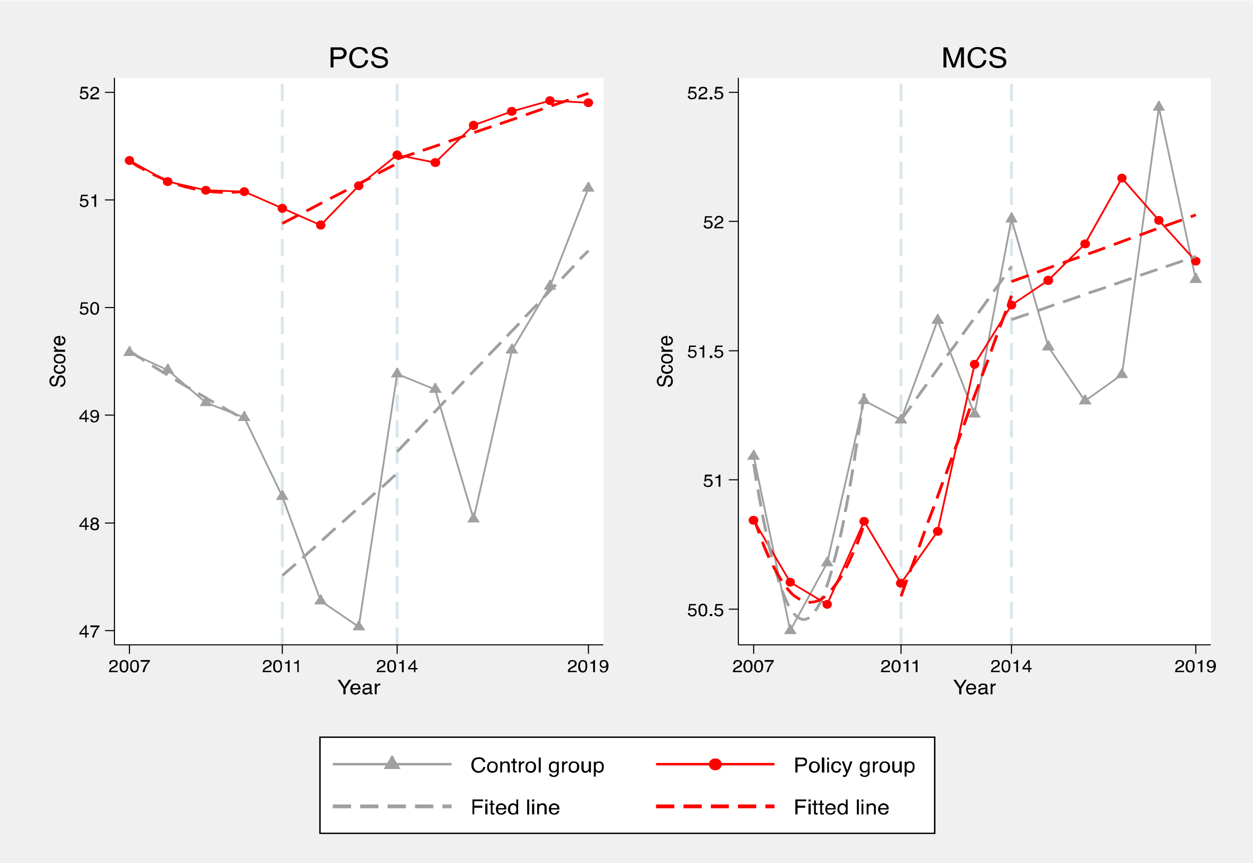

Notes: Sampling weights are applied to the calculation of mean PCS and MCS score estimates. The dashed lines indicate linear trends.

The appendix exhibit shows yearly trends in PCS and MCS scores separately for non-TRICARE adults (policy intervention group) and TRICARE beneficiaries (comparison group). Compared to TRICARE beneficiaries, non-TRICARE individuals always had higher year-to-year mean PCS scores. Both populations exhibited similarly-decreasing year-to-year trends in the pre-ACA period (2007-2010). There were overall upward trends in the post-ACA period, somewhat steeper for TRICARE beneficiaries. For both TRICARE and non-TRICARE individuals, mean MCS scores had more or less similar yearly trends with fluctuations over the study period; exhibited overall U-shaped trends in the pre-ACA period and increasing trends in the post-ACA period; and were higher overall in long-term post-ACA period than the immediate short-term post-ACA period.

Appendix C. Falsification regression analyses for parallel trend assumption

C1. Conditional-mean DID regression models:

The parallel trend assumption (or common trend assumption) is the fundamental assumption underlying the difference-in-differences analysis. It refers to the same trend in an outcome over time in the pre-policy time period (ie, before ACA implementation) between the policy and comparison groups. We performed two different regression-based falsification analyses to test whether the parallel trend assumption is satisfied in our data.

First, we estimated the following model on the pre-ACA subsample:

(C.1) $h_{igt} = G(\beta_0 + \beta_1 \cdot policy_g + \beta_2 \cdot time_t + \beta_3 \cdot policy_g \times time_t + x'_{igt} \cdot \beta + \tau_t + \tau_t^2) + \varepsilon_{igt}$

Here, the variable $time$ is a time trend and its coefficient β_2 measures a time trend for the control group in the pre-ACA period. The coefficient on the interaction term of policy group indicator and time trend β_3 measures an incremental effect of time trend for the policy group and should be insignificant if the conditional common trends assumption is satisfied.

Second, we examined the effects of placebo policies only using data for the pre-ACA period (years 2007-2010). We tested the effect of a placebo policy period #1 ($pp1$) that takes the value of 1 for year 2009 or later and 0 for years 2007 and 2008 and also another placebo policy period #2 ($pp2$) that takes the value of 1 for year 2008 or later and 0 for year 2007. Because our findings always remained robust, we only report results from the reference model below but other results are also available upon request. We estimated the following empirical models:

(C.2) $h_{igt} = G(\beta_0 + \beta_1 \cdot policy_g + \beta_2 \cdot pp1_t + \beta_3 \cdot policy_g \times pp1_t + x'_{igt} \cdot \beta + \tau_t + \tau_t^2) + \varepsilon_{igt}$

(C.3) $h_{igt} = G(\beta_0 + \beta_1 \cdot policy_g + \beta_2 \cdot pp2_t + \beta_3 \cdot policy_g \times pp2_t + x'_{igt} \cdot \beta + \tau_t + \tau_t^2) + \varepsilon_{igt}$

Here h_{igt} refers to the PSC or MCS score of individual i of the policy/control group g in year t. G is a function that links the explanatory variables to the outcome. The "$policy$" variable is the indicator for the policy group of the non-elderly, non-Tricare adult US population with the comparison group of non-elderly adult Tricare beneficiaries. The "$pp1$" and "$pp2$" variables indicate the placebo post-ACA implementation periods (years 2009-2010 and years 2008-2010, respectively). The interaction terms "$policy \times pp1$" and "$policy \times pp2$" are of primary interest and their coefficient "β_3" measures incremental changes in PCS and MCS scores attributable to ACA, which should be statistically insignificant under the conditional common trend assumption.

x' is a vector of covariates including age, sex, race/ethnicity (Black, all other race, and Hispanic; reference: non-Hispanic White), marital status (widowed, divorced, separated, and single; reference: married), census region of residence (northeast, mid-west, and west; reference: south), education (high school graduate, college graduate, and graduate school; reference: < high school), and family income (near poor, low-income, middle-income, high-income; reference: poor). We also controlled for the quadratic time trends.

Appendix Table C1. Analysis of parallel trends in PCS and MCS scores: main coefficient estimates ($\hat{\beta}_3$).

Falsification analysis	PCS score	MCS score
Eq. C.1: Time trends separately for the policy and control groups on the pre-ACA subsample	0.27 (0.39)	0.20 (0.31)
Eq. C.2: Placebo policy #1 on the pre-ACA subsample	0.59 (0.86)	0.50 (0.71)
Eq. C.3: Placebo policy #2 on the pre-ACA subsample	–0.40 (1.26)	0.43 (1.65)

Notes: Standard errors in parentheses.

C2. Percentile-specific simultaneous-quantile difference-in-differences regression models

We also estimated the following quantile regression models on the pre-ACA subsample:

(C.4) $Q_q(h_{igt}) = \beta_0 + \beta_1 \cdot policy_g + \beta_2 \cdot time_t + \beta_3 \cdot policy_g \times time_t + x'_{igt} \cdot \beta + \tau_t + \tau_t^2 + F^{-1}_{\varepsilon_{igt}}(q)$

(C.5) $Q_q(h_{igt}) = \beta_0 + \beta_1 \cdot policy_g + \beta_2 \cdot pp1_t + \beta_3 \cdot policy_g \times pp1_t + x'_{igt} \cdot \beta + \tau_t + \tau_t^2 + F^{-1}_{\varepsilon_{igt}}(q)$

(C.6) $Q_q(h_{igt}) = \beta_0 + \beta_1 \cdot policy_g + \beta_2 \cdot pp2_t + \beta_3 \cdot policy_g \times pp2_t + x'_{igt} \cdot \beta + \tau_t + \tau_t^2 + F^{-1}_{\varepsilon_{igt}}(q)$

As shown result tables below, none of the main coefficients was statistically significant, which support the parallel trend assumption underlying our difference-in-differences approach.

Appendix Table C2. Analysis of parallel trends in PCS and MCS scores: main coefficient estimates ($\hat{\beta}_3$) across quantiles (q):

Falsification analysis	q=.20	q=.40	q=.60	q=.80
Outcome: PCS score				
Eq. C.4: Time trends separately for the policy and control groups on the pre-ACA subsample	0.6184 (0.5185)	–0.1150 (0.3167)	–0.0116 (0.1895)	0.1745 (0.1262)
Eq. C.5: Placebo policy #1 on the pre–ACA subsample	1.8269 (1.1432)	–0.3046 (0.6988)	–0.1366 (0.4202)	0.3095 (0.2784)
Eq. C.6: Placebo policy #2 on the pre–ACA subsample	0.8507 (1.3947)	–0.2439 (0.8553)	0.0671 (0.5099)	0.0200 (0.3422)
Outcome: MCS score				
Eq. C.4: Time trends separately for the policy and control groups on the pre-ACA subsample	0.2356 (0.5060)	0.0961 (0.3818)	–0.0678 (0.2709)	–0.1993 (0.2362)
Eq. C.5: Placebo policy #1 on the pre–ACA subsample	–0.0643 (1.1235)	–0.2730 (0.8526)	–0.3657 (0.6055)	–0.8098 (0.5156)
Eq. C.6: Placebo policy #2 on the pre–ACA subsample	2.1433 (1.3598)	0.8473 (1.0339)	–0.1500 (0.7337)	–0.2789 (0.6358)

Notes: Standard errors in parentheses.

Appendix D. Effects of ACA on PCS and MCS scores: Conditional-mean DID models.

Variables	Overall effect (2011-2019)		Short- (2011-2013)/long–term (2014-2019) effects	
	PCS score	MCS score	PCS score	MCS score
Main explanatory variables				
Policy intervention×Post (2011-19)	0.577<br>(0.535)	0.635<br>(0.518)		
Policy intervention ×Short-term post (2011-13)			1.359*<br>(0.660)	0.240<br>(0.643)
Policy intervention ×Long-term post (2014-19)			0.251<br>(0.583)	0.807<br>(0.556)
Policy intervention group	0.816<br>(0.440)	–0.408<br>(0.488)	0.815<br>(0.440)	–0.412<br>(0.488)
Post-ACA period (2011-19)	–0.746<br>(0.550)	–0.691<br>(0.508)		
Short-term post-ACA period (2011-13)			–1.490*<br>(0.675)	–0.228<br>(0.627)
Long-term post-ACA period (2014-19)			–0.273<br>(0.599)	–0.342<br>(0.569)
Covariates				
Race/ethnicity (reference: White)				
Non–Hispanic Black	–0.596***<br>(0.095)	1.895***<br>(0.104)	–0.596***<br>(0.095)	1.893***<br>(0.104)
All Other Race	0.436***<br>(0.107)	1.201***<br>(0.114)	0.436***<br>(0.107)	1.197***<br>(0.114)
Hispanic	1.432***<br>(0.104)	1.998***<br>(0.101)	1.433***<br>(0.104)	1.998***<br>(0.101)
Age	–0.221***<br>(0.003)	–0.017***<br>(0.003)	–0.221***<br>(0.003)	–0.017***<br>(0.003)
Female	–0.114*<br>(0.055)	–1.487***<br>(0.055)	–0.115*<br>(0.055)	–1.487***<br>(0.055)
Marital status (reference: Married)				
Widow	–3.348***<br>(0.306)	–2.668***<br>(0.279)	–3.347***<br>(0.306)	–2.670***<br>(0.280)
Divorced	–1.825***<br>(0.112)	–2.198***<br>(0.108)	–1.823***<br>(0.112)	–2.197***<br>(0.108)
Separated	–1.414***<br>(0.199)	–3.357***<br>(0.233)	–1.412***<br>(0.199)	–3.357***<br>(0.232)
Single	3.061***<br>(0.075)	–0.697***<br>(0.082)	3.061***<br>(0.075)	–0.697***<br>(0.082)
Census region (reference: South)				
Northeast	0.502***	–0.043	0.503***	–0.043

	(0.116)	(0.132)	(0.116)	(0.133)
Midwest	–0.007	–0.300**	–0.006	–0.300**
	(0.114)	(0.107)	(0.114)	(0.107)
West	0.232*	–0.469***	0.233*	–0.469***
	(0.111)	(0.102)	(0.111)	(0.102)
Education (reference: < high school)				
High school	0.328***	0.461***	0.329***	0.465***
	(0.086)	(0.097)	(0.086)	(0.098)
College	1.502***	0.269**	1.503***	0.272**
	(0.091)	(0.094)	(0.091)	(0.094)
> college	1.999***	0.357*	2.001***	0.368**
	(0.129)	(0.140)	(0.129)	(0.140)
Unemployed	–4.905***	–2.119***	–4.904***	–2.114***
	(0.110)	(0.087)	(0.111)	(0.087)
Family income (reference: Poor)				
Near poor (100% to < 125% FPL)	0.127	0.881***	0.126	0.882***
	(0.167)	(0.187)	(0.167)	(0.187)
Low (125% to < 200% FPL)	1.056***	1.592***	1.056***	1.593***
	(0.121)	(0.133)	(0.121)	(0.133)
Middle (200% to < 400% FPL)	2.161***	2.734***	2.161***	2.734***
	(0.114)	(0.118)	(0.114)	(0.118)
High (≥ 400% FPL)	2.982***	3.838***	2.981***	3.835***
	(0.117)	(0.122)	(0.117)	(0.123)
Time trends				
t	–0.034	0.223***	–0.049	0.173**
	(0.058)	(0.057)	(0.056)	(0.057)
t^2	0.006	–0.007*	0.005	–0.007*
	(0.003)	(0.003)	(0.003)	(0.003)
Intercept	47.9***	49.1***	48.0***	49.2***
	(0.481)	(0.536)	(0.479)	(0.533)
n	215,572	215,663	215,572	215,663

Notes: Standard errors in parentheses. * $p < 0.05$, ** $p < 0.01$, *** $p < 0.001$

Appendix E. Percentile-specific overall (2011-2019) effect of ACA on PCS and MCS scores at deciles: Simultaneous-quantile DID estimates.

Panel A. Outcome: PCS score

Variables	Quantile (q)								
	q=.10	q=.20	q=.30	q=.40	q=.50	q=.60	q=.70	q=.80	q=.90
Main explanatory variables									
Policy intervention×Post (2011-19)	0.560	1.415*	0.834	0.773*	0.230	–0.013	0.188	0.028	–0.037
	(0.906)	(0.631)	(0.634)	(0.389)	(0.286)	(0.221)	(0.185)	(0.131)	(0.183)
Policy group	3.712***	2.451***	1.657**	0.968**	0.766**	0.674***	0.262	0.274*	0.370*
	(0.812)	(0.491)	(0.576)	(0.302)	(0.239)	(0.199)	(0.190)	(0.114)	(0.163)
Policy intervention group	–1.060	–1.794**	–1.122	–1.006*	–0.326	–0.103	–0.262	–0.107	–0.014
	(0.886)	(0.637)	(0.649)	(0.397)	(0.290)	(0.225)	(0.184)	(0.133)	(0.183)
Covariates									
Race/ethnicity (reference: White)									
Non-Hispanic Black	–0.060	–0.667***	–0.828***	–0.748***	–0.645***	–0.603***	–0.623***	–0.633***	–0.741***
	(0.144)	(0.106)	(0.083)	(0.065)	(0.044)	(0.034)	(0.028)	(0.027)	(0.041)
All Other Race	1.181***	0.559***	0.173*	–0.002	–0.071	–0.178***	–0.275***	–0.320***	–0.407***
	(0.160)	(0.095)	(0.076)	(0.061)	(0.044)	(0.031)	(0.031)	(0.035)	(0.045)
Hispanic	2.637***	1.566***	0.855***	0.426***	0.119**	–0.095***	–0.255***	–0.283***	–0.270***
	(0.128)	(0.089)	(0.065)	(0.051)	(0.039)	(0.028)	(0.024)	(0.027)	(0.041)
Age	–0.468***	–0.376***	–0.288***	–0.212***	–0.151***	–0.110***	–0.080***	–0.063***	–0.066***
	(0.004)	(0.002)	(0.002)	(0.002)	(0.001)	(0.001)	(0.001)	(0.001)	(0.001)
Female	–1.778***	–1.337***	–1.008***	–0.693***	–0.449***	–0.267***	–0.104***	0.026	0.124***
	(0.099)	(0.061)	(0.054)	(0.039)	(0.028)	(0.021)	(0.017)	(0.016)	(0.026)
Family income (reference: Poor)									
Near poor (100% to < 125% FPL)	1.893***	2.010***	1.960***	1.583***	0.972***	0.558***	0.269***	0.164***	0.092
	(0.294)	(0.266)	(0.203)	(0.119)	(0.094)	(0.062)	(0.047)	(0.044)	(0.068)
Low (125% to < 200% FPL)	4.503***	4.694***	3.875***	2.766***	1.765***	1.025***	0.609***	0.394***	0.243***
	(0.198)	(0.158)	(0.130)	(0.095)	(0.073)	(0.051)	(0.037)	(0.033)	(0.046)
Middle (200% to < 400% FPL)	7.830***	7.355***	5.790***	4.064***	2.618***	1.649***	1.055***	0.739***	0.505***
	(0.166)	(0.136)	(0.103)	(0.075)	(0.056)	(0.040)	(0.032)	(0.029)	(0.037)

High (≥400% FPL)	12.241***	10.309***	7.943***	5.680***	3.807***	2.571***	1.757***	1.281***	0.912***
	(0.168)	(0.142)	(0.102)	(0.077)	(0.061)	(0.045)	(0.035)	(0.031)	(0.039)
Time trends									
t	0.166	0.125*	0.141**	0.107**	0.041	0.045*	0.036*	0.036*	0.024
	(0.096)	(0.054)	(0.050)	(0.037)	(0.028)	(0.021)	(0.018)	(0.018)	(0.027)
t^2	0.003	0.001	–0.002	–0.002	0.001	–0.002	–0.003**	–0.004***	–0.005***
	(0.006)	(0.003)	(0.003)	(0.002)	(0.002)	(0.001)	(0.001)	(0.001)	(0.002)
Intercept	46.122***	50.988***	53.543***	55.520***	56.692***	57.497***	58.443***	59.082***	60.854***
	(0.886)	(0.502)	(0.584)	(0.316)	(0.269)	(0.219)	(0.218)	(0.137)	(0.184)

Notes: Standard errors in parentheses. * $p < 0.05$, ** $p < 0.01$, *** $p < 0.001$

Panel B. Outcome: MCS score

Variable	Quantile (q)								
	q=.10	q=.20	q=.30	q=.40	q=.50	q=.60	q=.70	q=.80	q=.90
Main explanatory variables									
Policy intervention×Post (2011-19)	0.836	1.389	1.506**	1.207*	0.676	0.616*	0.339	0.494	0.085
	(1.033)	(0.945)	(0.545)	(0.488)	(0.415)	(0.284)	(0.245)	(0.272)	(0.186)
Policy intervention group	0.608	0.057	–0.809	–0.801	–0.585	–0.475*	–0.380	–0.430	–0.184
	(0.913)	(0.812)	(0.438)	(0.424)	(0.342)	(0.227)	(0.213)	(0.222)	(0.170)
Post-ACA period (2011-19)	–0.915	–1.502	–1.609**	–1.345**	–0.804	–0.631*	–0.341	–0.383	–0.132
	(1.010)	(0.947)	(0.555)	(0.499)	(0.438)	(0.301)	(0.257)	(0.279)	(0.189)
Covariates									
Race/ethnicity (reference: White)									
Non–Hispanic Black	2.597***	2.135***	1.777***	1.692***	1.616***	1.386***	1.262***	1.774***	1.821***
	(0.147)	(0.117)	(0.105)	(0.076)	(0.057)	(0.053)	(0.045)	(0.053)	(0.037)
All Other Race	2.277***	1.577***	1.157***	1.032***	1.007***	0.847***	0.761***	1.329***	1.729***
	(0.155)	(0.132)	(0.095)	(0.075)	(0.079)	(0.058)	(0.042)	(0.061)	(0.038)
Hispanic	3.116***	2.070***	1.585***	1.487***	1.494***	1.540***	1.688***	2.404***	2.061***
	(0.129)	(0.104)	(0.088)	(0.071)	(0.063)	(0.046)	(0.048)	(0.039)	(0.033)
Age	–0.071***	–0.080***	–0.070***	–0.055***	–0.040***	–0.027***	–0.014***	–0.007***	–0.002*
	(0.004)	(0.003)	(0.003)	(0.002)	(0.002)	(0.002)	(0.001)	(0.001)	(0.001)
Female	–2.406***	–2.306***	–2.163***	–1.937***	–1.756***	–1.455***	–1.052***	–0.995***	–0.435***
	(0.099)	(0.075)	(0.063)	(0.045)	(0.043)	(0.037)	(0.032)	(0.037)	(0.034)
Family income (reference: Poor)									
Near poor (100% to < 125% FPL)	2.070***	2.087***	2.151***	1.892***	1.388***	1.110***	0.589***	0.302***	0.081
	(0.204)	(0.192)	(0.204)	(0.158)	(0.125)	(0.118)	(0.106)	(0.080)	(0.043)
Low (125% to < 200% FPL)	4.011***	3.706***	3.756***	3.261***	2.596***	1.972***	1.091***	0.474***	0.051
	(0.171)	(0.160)	(0.149)	(0.126)	(0.110)	(0.092)	(0.061)	(0.055)	(0.028)
Middle (200% to < 400% FPL)	6.730***	6.344***	6.031***	5.113***	3.945***	2.709***	1.449***	0.632***	0.098***
	(0.146)	(0.130)	(0.119)	(0.096)	(0.084)	(0.080)	(0.053)	(0.047)	(0.022)
High (≥400% FPL)	9.974***	9.411***	8.357***	6.820***	5.160***	3.496***	1.866***	0.912***	0.173***
	(0.147)	(0.121)	(0.119)	(0.104)	(0.083)	(0.078)	(0.057)	(0.058)	(0.030)
Time trends									
t	0.212*	0.274**	0.270***	0.308***	0.271***	0.194***	0.107***	0.159***	0.307***

	(0.108)	(0.089)	(0.068)	(0.055)	(0.054)	(0.037)	(0.026)	(0.029)	(0.022)
t^2	0.003	–0.001	–0.002	–0.007*	–0.007*	–0.005*	–0.001	–0.008***	–0.026***
	(0.007)	(0.005)	(0.004)	(0.003)	(0.003)	(0.002)	(0.001)	(0.002)	(0.001)
Intercept	32.286***	39.810***	45.008***	48.271***	51.125***	53.796***	56.138***	57.880***	59.982***
	(0.962)	(0.845)	(0.480)	(0.456)	(0.373)	(0.254)	(0.221)	(0.240)	(0.182)

Notes: Standard errors in parentheses. * $p < 0.05$, ** $p < 0.01$, *** $p < 0.001$

Appendix F. Percentile-specific short-term (2011-2013) vs. long-term (2014-2019) effects of ACA on PCS and MCS scores at deciles: Simultaneous-quantile DID estimates.

Panel A. Outcome: PCS score

Variable	Quantile (q)								
	q=.10	q=.20	q=.30	q=.40	q=.50	q=.60	q=.70	q=.80	q=.90
Main explanatory variables									
Policy intervention×Short-term post (2011-13)	0.924	1.779	1.039	1.086	0.435	0.132	0.290	0.021	0.097
	(1.139)	(0.983)	(1.249)	(0.609)	(0.456)	(0.336)	(0.278)	(0.215)	(0.244)
Policy intervention×Long-term post (2014-19)	0.405	1.407*	0.744	0.478	0.197	–0.139	0.180	0.054	–0.060
	(0.962)	(0.705)	(0.638)	(0.379)	(0.279)	(0.236)	(0.235)	(0.159)	(0.217)
Short-term post-ACA period (2011-13)	–1.379	–2.061*	–1.195	–1.213*	–0.506	–0.138	–0.230	0.054	0.048
	(1.144)	(0.980)	(1.255)	(0.605)	(0.447)	(0.329)	(0.272)	(0.208)	(0.242)
Long-term post-ACA period (2014-19)	–0.443	–1.231	–0.599	–0.406	–0.104	0.320	0.025	0.132	0.322
	(0.993)	(0.723)	(0.653)	(0.401)	(0.290)	(0.239)	(0.228)	(0.163)	(0.222)
Covariates									
Race/ethnicity (reference: White)									
Non–Hispanic Black	–0.086	–0.674***	–0.836***	–0.747***	–0.652***	–0.603***	–0.615***	–0.626***	–0.739***
	(0.161)	(0.101)	(0.079)	(0.059)	(0.044)	(0.033)	(0.026)	(0.028)	(0.033)
All Other Race	1.164***	0.548***	0.171*	–0.001	–0.077	–0.179***	–0.275***	–0.316***	–0.398***
	(0.155)	(0.090)	(0.083)	(0.066)	(0.049)	(0.034)	(0.031)	(0.029)	(0.046)
Hispanic	2.624***	1.549***	0.855***	0.431***	0.110**	–0.095***	–0.250***	–0.279***	–0.267***
	(0.110)	(0.067)	(0.056)	(0.045)	(0.036)	(0.026)	(0.021)	(0.023)	(0.036)
Age	–0.468***	–0.376***	–0.289***	–0.212***	–0.151***	–0.110***	–0.080***	–0.063***	–0.066***
	(0.004)	(0.003)	(0.002)	(0.002)	(0.002)	(0.001)	(0.001)	(0.001)	(0.001)
Female	–1.762***	–1.347***	–1.002***	–0.694***	–0.444***	–0.265***	–0.105***	0.023	0.121***
	(0.100)	(0.068)	(0.053)	(0.037)	(0.029)	(0.024)	(0.018)	(0.019)	(0.027)
Family income (reference: Poor)									
Near poor (100% to < 125% FPL)	1.934***	2.002***	1.976***	1.589***	0.977***	0.559***	0.270***	0.167**	0.092
	(0.225)	(0.212)	(0.195)	(0.119)	(0.098)	(0.061)	(0.047)	(0.052)	(0.076)
Low (125% to < 200% FPL)	4.543***	4.675***	3.877***	2.767***	1.765***	1.028***	0.615***	0.391***	0.235***
	(0.217)	(0.163)	(0.117)	(0.096)	(0.073)	(0.051)	(0.038)	(0.039)	(0.051)
Middle (200% to < 400% FPL)	7.857***	7.344***	5.791***	4.067***	2.620***	1.650***	1.055***	0.734***	0.503***
	(0.158)	(0.121)	(0.097)	(0.081)	(0.057)	(0.042)	(0.031)	(0.030)	(0.040)

High (≥400% FPL)	12.269***	10.291***	7.955***	5.682***	3.809***	2.573***	1.755***	1.276***	0.904***
	(0.161)	(0.125)	(0.096)	(0.087)	(0.063)	(0.045)	(0.033)	(0.030)	(0.043)
Time trends									
t	0.160***	0.081***	0.067***	0.051***	0.026*	–0.016	–0.040***	–0.053***	–0.083***
	(0.039)	(0.022)	(0.016)	(0.014)	(0.011)	(0.008)	(0.007)	(0.006)	(0.008)
Intercept	46.133***	51.239***	53.700***	55.621***	56.739***	57.640***	58.640***	59.290***	61.107***
	(0.786)	(0.531)	(0.549)	(0.279)	(0.235)	(0.222)	(0.207)	(0.130)	(0.178)

Notes: Standard errors in parentheses. * $p < 0.05$, ** $p < 0.01$, *** $p < 0.001$

Panel B. Outcome: MCS score

Variable	Quantile (q)								
	q=.10	q=.20	q=.30	q=.40	q=.50	q=.60	q=.70	q=.80	q=.90
Main explanatory variables									
Policy intervention×Short-term post (2011-13)	–0.919<br>(1.068)	0.845<br>(1.096)	1.310<br>(0.721)	0.929<br>(0.606)	0.509<br>(0.510)	0.530<br>(0.383)	0.282<br>(0.274)	0.297<br>(0.348)	0.023<br>(0.228)
Policy intervention×Long-term post (2014-19)	1.277<br>(1.057)	1.808<br>(1.025)	1.654**<br>(0.628)	1.330*<br>(0.543)	0.818<br>(0.468)	0.716*<br>(0.307)	0.420<br>(0.261)	0.565<br>(0.290)	0.200<br>(0.167)
Short-term post-ACA period (2011-13)	1.078<br>(1.069)	–0.913<br>(1.065)	–1.333<br>(0.711)	–0.943<br>(0.605)	–0.497<br>(0.515)	–0.409<br>(0.378)	–0.185<br>(0.272)	–0.082<br>(0.339)	0.131<br>(0.223)
Long-term post-ACA period (2014-19)	–0.236<br>(1.062)	–1.621<br>(0.995)	–1.369*<br>(0.656)	–0.898<br>(0.579)	–0.283<br>(0.489)	–0.133<br>(0.316)	0.125<br>(0.261)	0.272<br>(0.291)	0.465**<br>(0.166)
Covariates									
Race/ethnicity (reference: White)									
Non–Hispanic Black	2.602***<br>(0.146)	2.143***<br>(0.122)	1.767***<br>(0.107)	1.700***<br>(0.081)	1.615***<br>(0.059)	1.390***<br>(0.048)	1.264***<br>(0.039)	1.774***<br>(0.043)	1.868***<br>(0.036)
All Other Race	2.266***<br>(0.163)	1.585***<br>(0.124)	1.152***<br>(0.106)	1.027***<br>(0.076)	1.007***<br>(0.068)	0.865***<br>(0.056)	0.777***<br>(0.048)	1.295***<br>(0.071)	1.780***<br>(0.041)
Hispanic	3.086***<br>(0.120)	2.067***<br>(0.096)	1.583***<br>(0.076)	1.496***<br>(0.059)	1.494***<br>(0.052)	1.547***<br>(0.044)	1.682***<br>(0.047)	2.394***<br>(0.036)	2.042***<br>(0.030)
Age	–0.070***<br>(0.004)	–0.080***<br>(0.003)	–0.071***<br>(0.003)	–0.054***<br>(0.002)	–0.040***<br>(0.002)	–0.026***<br>(0.002)	–0.013***<br>(0.001)	–0.007***<br>(0.001)	–0.001<br>(0.001)
Female	–2.421***<br>(0.108)	–2.306***<br>(0.083)	–2.166***<br>(0.069)	–1.937***<br>(0.056)	–1.753***<br>(0.046)	–1.462***<br>(0.039)	–1.075***<br>(0.035)	–1.024***<br>(0.033)	–0.416***<br>(0.028)
Family income (reference: Poor)									
Near poor (100% to < 125% FPL)	2.090***<br>(0.225)	2.076***<br>(0.184)	2.153***<br>(0.196)	1.883***<br>(0.139)	1.424***<br>(0.123)	1.107***<br>(0.117)	0.584***<br>(0.083)	0.358***<br>(0.079)	0.069<br>(0.037)
Low (125% to < 200% FPL)	4.067***<br>(0.144)	3.683***<br>(0.130)	3.759***<br>(0.127)	3.270***<br>(0.105)	2.592***<br>(0.092)	1.968***<br>(0.080)	1.085***<br>(0.065)	0.511***<br>(0.058)	0.041<br>(0.030)
Middle (200% to < 400% FPL)	6.786***<br>(0.147)	6.335***<br>(0.125)	6.030***<br>(0.121)	5.102***<br>(0.092)	3.951***<br>(0.082)	2.719***<br>(0.071)	1.458***<br>(0.056)	0.679***<br>(0.052)	0.082**<br>(0.028)
High (≥400% FPL)	10.005***<br>(0.154)	9.408***<br>(0.110)	8.351***<br>(0.111)	6.821***<br>(0.089)	5.158***<br>(0.078)	3.493***<br>(0.067)	1.833***<br>(0.060)	0.941***<br>(0.054)	0.148***<br>(0.027)
Time trends									
t	0.074<br>(0.114)	0.248**<br>(0.084)	0.237***<br>(0.064)	0.242***<br>(0.049)	0.191***<br>(0.046)	0.126***<br>(0.037)	0.056*<br>(0.022)	0.098***<br>(0.022)	0.188***<br>(0.018)

t^2	0.004	–0.002	–0.003	–0.007*	–0.006*	–0.005*	–0.002	–0.009***	–0.024***
	(0.006)	(0.005)	(0.004)	(0.003)	(0.003)	(0.002)	(0.001)	(0.001)	(0.001)
Intercept	32.584***	39.892***	45.136***	48.394***	51.360***	53.958***	56.267***	58.037***	60.338***
	(0.932)	(0.882)	(0.554)	(0.460)	(0.356)	(0.239)	(0.191)	(0.250)	(0.161)

Notes: Standard errors in parentheses. * $p < 0.05$, ** $p < 0.01$, *** $p < 0.001$

Appendix G. Supplemental/robustness analysis

Appendix Table G.1. Conditional-mean difference-in-differences regression models: main estimates.

Robustness tests and variables	Overall effect (2011-2019)		Short- (2011-2013)/long–term (2014-2019) effects	
	PCS	MCS	PCS	MCS
Main DID estimates				
**Policy intervention×Post (2011-19)**	**0.577**	**0.635**		
	**(0.535)**	**(0.518)**		
**Policy intervention ×Short-term post (2011-13)**			**1.359***	**0.240**
			**(0.660)**	**(0.643)**
**Policy intervention ×Long-term post (2014-19)**			**0.251**	**0.807**
			**(0.583)**	**(0.556)**
Adjust for inverse-probability-weights				
Policy intervention×Post-ACA (2011-19)	0.882<br>(0.530)	1.049<br>(0.600)		
Policy intervention×Short-term post-ACA (2011-13)			1.611*<br>(0.659)	0.323<br>(0.727)
Policy intervention×Long-term post-ACA (2014-19)			0.533<br>(0.561)	1.430*<br>(0.644)
Drop uninsured				
Policy intervention×Post-ACA (2011-19)	0.675<br>(0.535)	0.559<br>(0.516)		
Policy intervention×Short-term post-ACA (2011-13)			1.405*<br>(0.661)	0.222<br>(0.635)
Policy intervention×Long-term post-ACA (2014-19)			0.371<br>(0.580)	0.705<br>(0.554)
Include group-specific time trends				
Policy intervention×Post-ACA (2011-19)	1.814*<br>(0.831)	0.158<br>(0.771)		
Policy intervention×Short-term post-ACA (2011-13)			1.711*<br>(0.848)	0.278<br>(0.763)
Policy intervention×Long-term post-ACA (2014-19)			1.123<br>(1.424)	0.885<br>(1.182)
Include time-trends specific to pre and post periods				
Policy intervention×Post-ACA (2011-19)	0.579<br>(0.536)	0.652<br>(0.517)		
Policy intervention×Short-term post-ACA (2011-13)			1.357*<br>(0.660)	0.251<br>(0.642)
			0.252	0.819

Policy intervention×Long-term post-ACA (2014-19)			(0.583)	(0.555)
Drop outliers				
Policy intervention×Post-ACA (2011-19)	0.577<br>(0.535)	0.635<br>(0.518)		
Policy intervention×Short-term post-ACA (2011-13)			1.351*<br>(0.660)	0.251<br>(0.642)
Policy intervention×Long-term post-ACA (2014-19)			0.242<br>(0.583)	0.819<br>(0.555)

Notes: Standard errors in parentheses. *p < 0.05. **p < 0.01, ***p < 0.001.

Appendix Figure G.1. Simultaneous-quantile difference-in-differences estimates: Overall effects of ACA (2011-2019)

- Include group-specific time trends

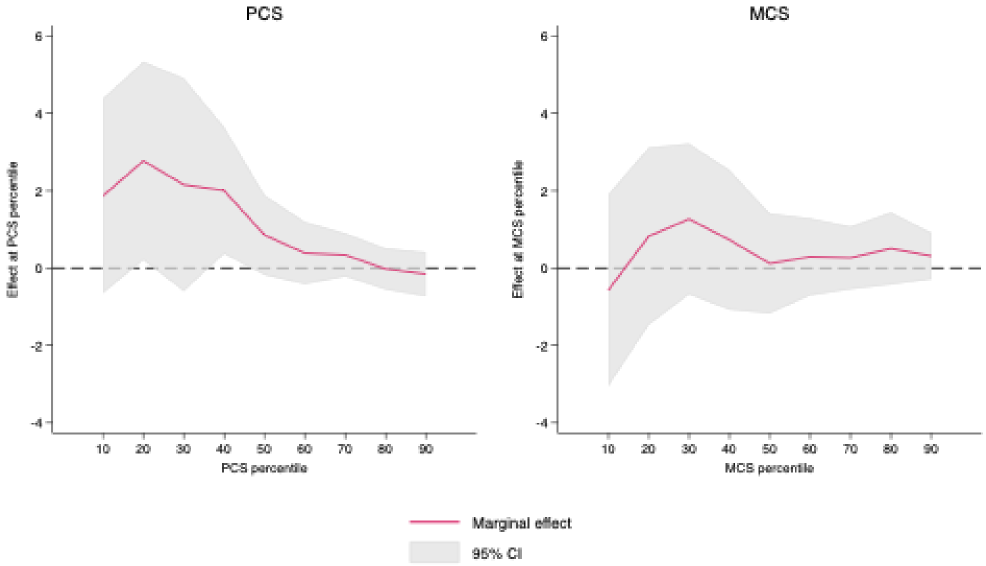

- Include time-trends specific to pre and post periods

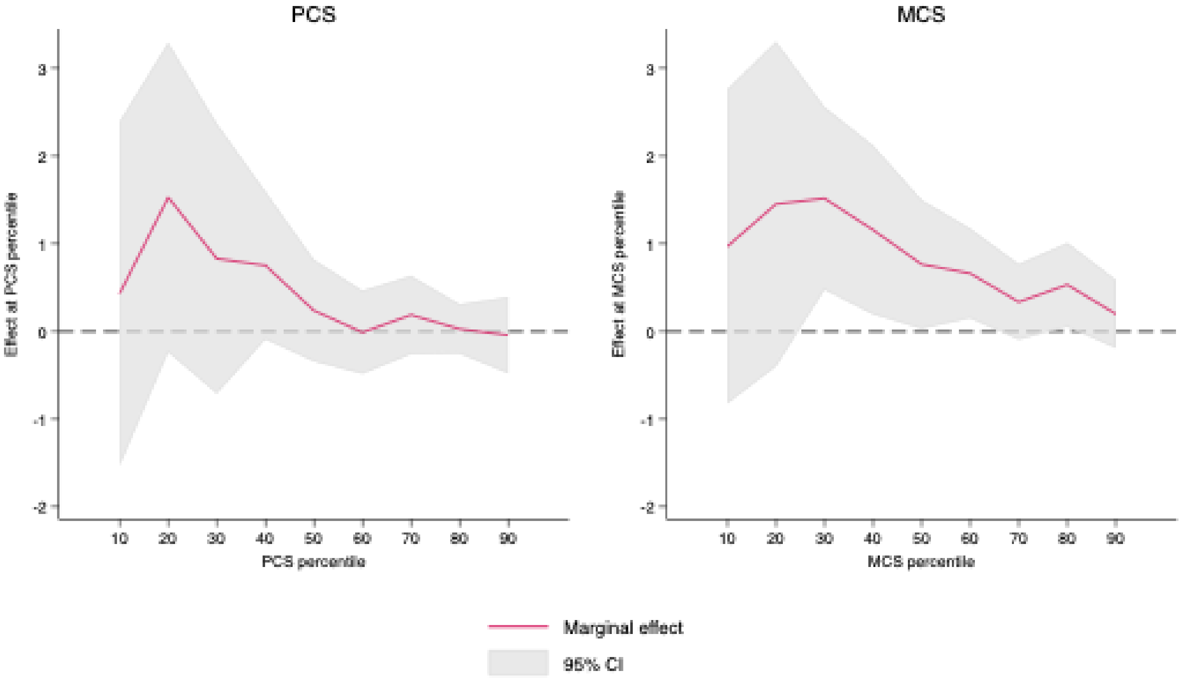

Drop outliers

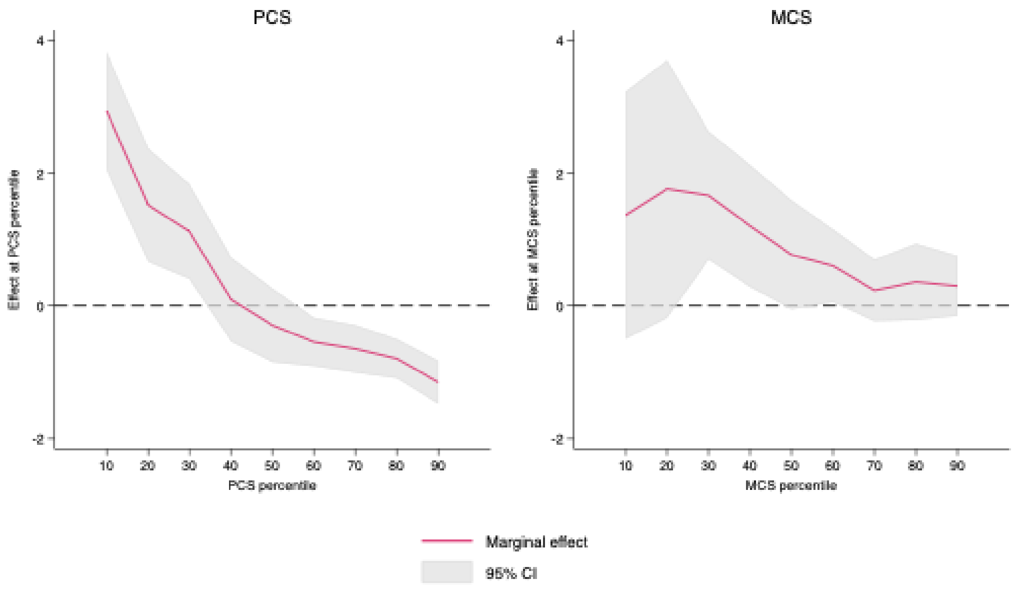

- Drop the uninsured

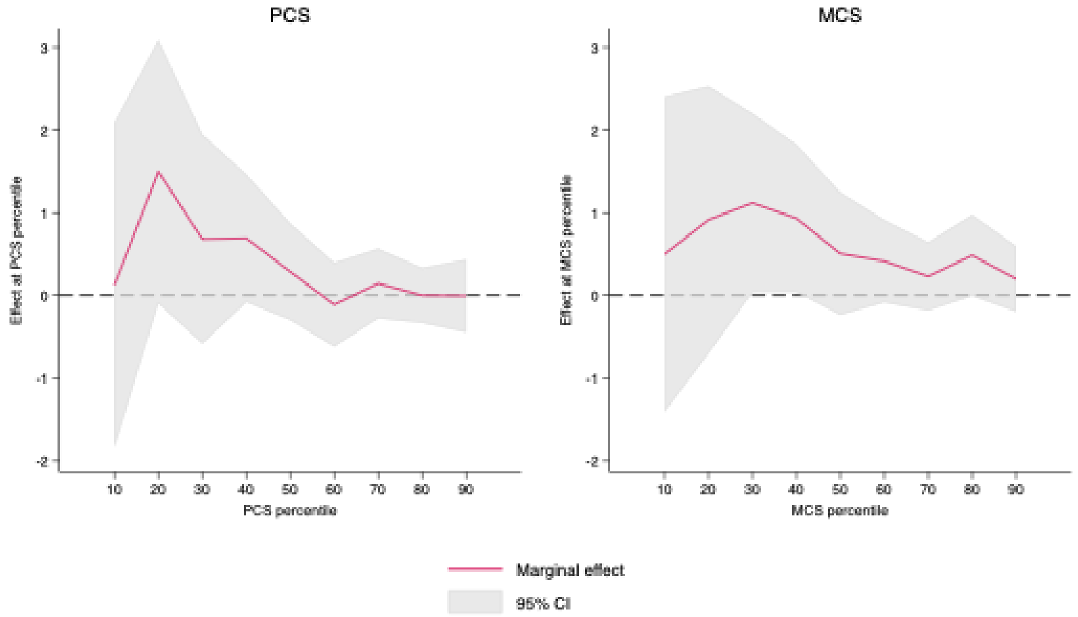

Appendix Figure G.2. Simultaneous-quantile difference-in-differences estimates: Short- (2011-2013) vs. long-term (2014-2019) effects of ACA

- Include group-specific time trends

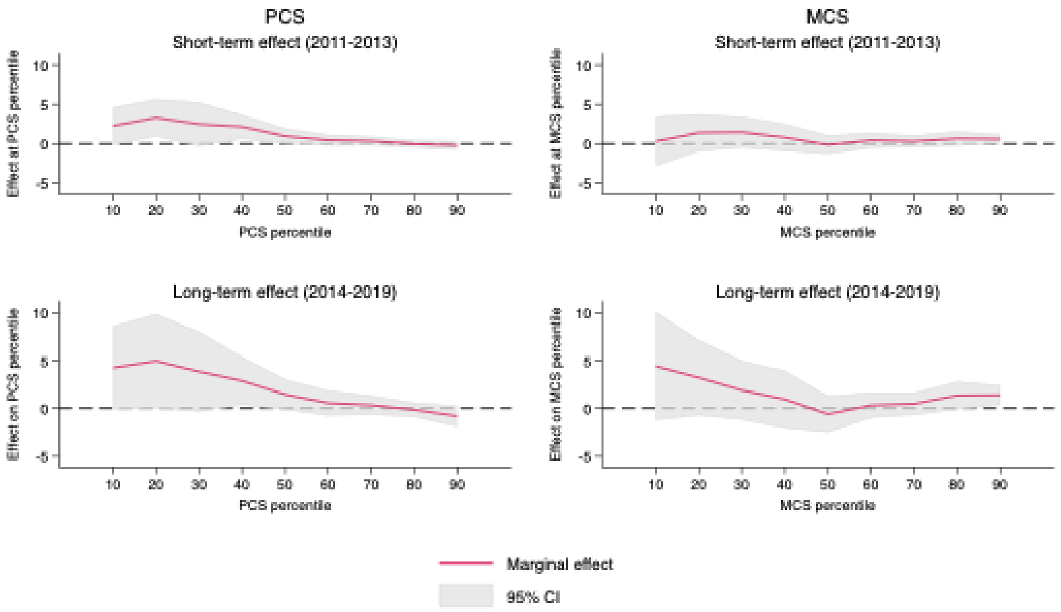

- Include time-trends specific to pre and post periods

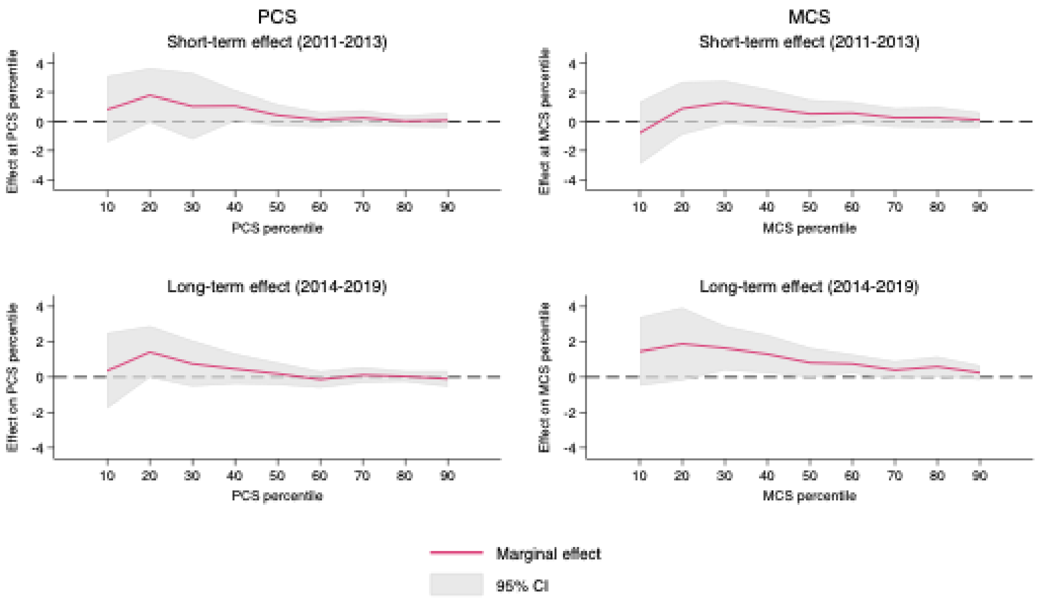

- Drop outliers

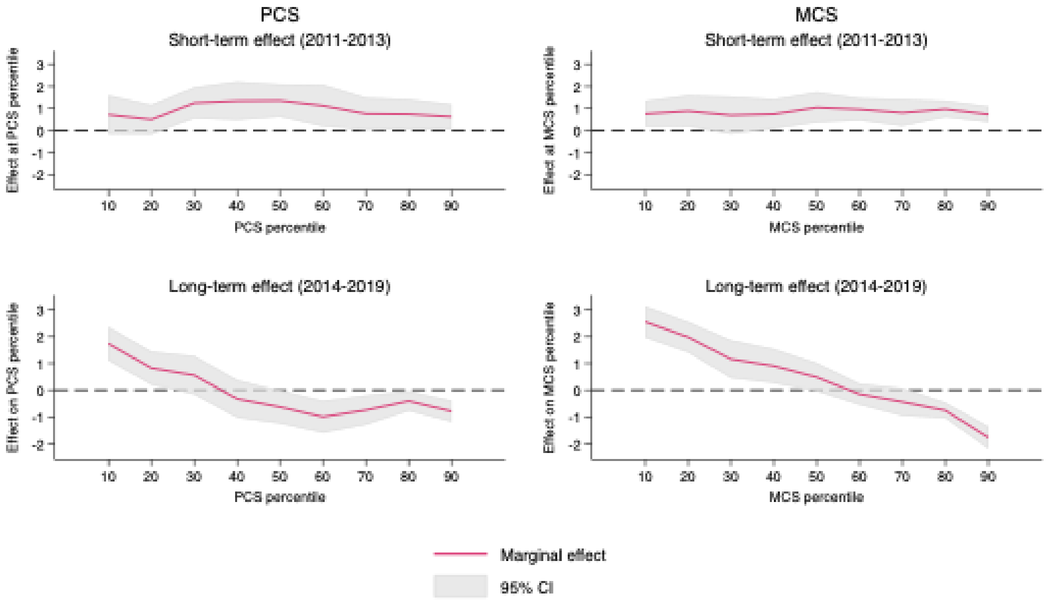

- Drop the uninsured

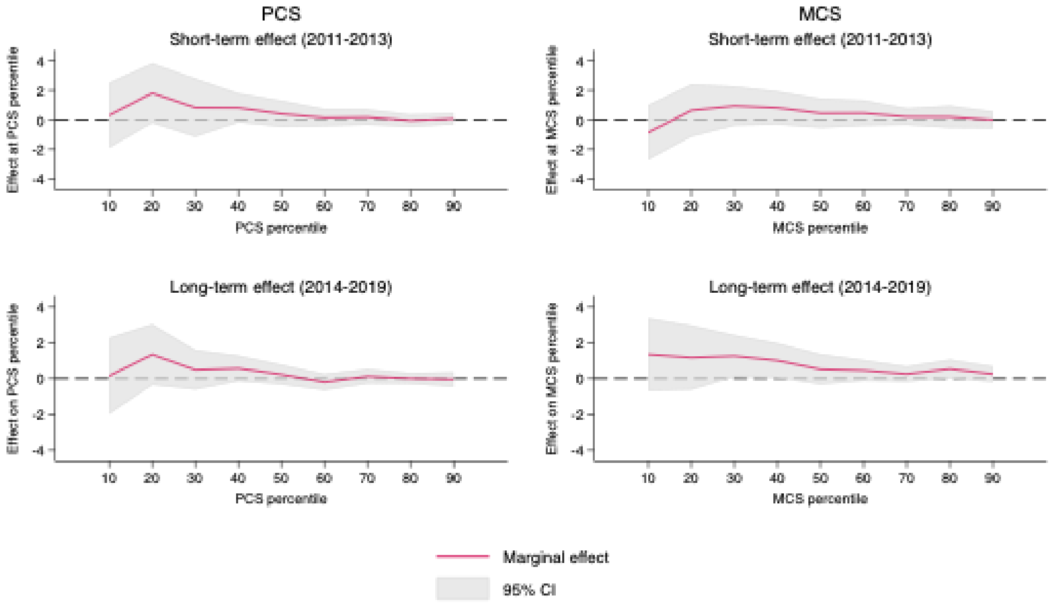

Appendix H. Inverse-Probability-Weighted Difference-in-Differences

The attribution of causality in DD on pooled cross-sectional data may be threatened by two factors: (a) a non-parallel evolution in the outcome between the policy intervention and control groups prior to policy intervention and (b) a different compositional evolution between the two groups. Stuart et al. (2014) showed that propensity score weights can be used to minimize bias due to selection observables in pooled cross-section DD analysis that occurs before and after policy change (by adjusting pre-post compositional differences) and also between policy intervention and control groups (by adjusting between-group compositional differences pre-parity).

The application of the technique is straightforward. We first estimated multinomial logistic regression models with covariates carefully selected not to be affected by the outcome measures:

$$ln\left(\frac{P(C=j)}{P(C=1)}\right) = X' \cdot \delta_j$$

where $\delta_j = (\delta_{j0}, \delta_{j1}, \delta_{j2}, \ldots)$. C is a categorical variable with four categories: $j = 1$ (Policy intervention group, Post-ACA), 2 (Policy intervention group, Pre-ACA), 3 (Control group, Post-ACA), and 4 (Control group, Pre-ACA). X' includes the covariates included in the main specification, as well as the interaction between age and sex and indicator for having a chronic condition. The model estimates the log-odds of choosing category j over category 1. We then obtain propensity scores by estimating the probability of each category j, relative to the base category, $j = 1$. For each individual i, four propensity scores, $(\sum_{k=1}^{4} p_k)_i$, are obtained:

$$p_j = P(C=j) = \frac{\exp\,(X' \cdot \delta_j)}{1 + \sum_{k=2}^{4} \exp\,(X' \cdot \delta_k)}, \qquad j = 2, 3, 4$$

$$p_1 = P(C=1) = \frac{1}{1 + \sum_{k=2}^{4} \exp\,(X' \cdot \delta_k)}, \qquad j = 1$$

Inverse-probability-weights (IPWs) were then constructed to make each of the four groups comparable to the base group, the policy intervention group in the pre-parity period. For each individual i, the weight is calculated as:

$$\text{IPW}_i = \frac{p_1}{p_j}$$

where p_1 is the estimated probability of being in the base category and p_j is the probability of being in the individual's actual category. As a result, individuals in the base category receives a weight of 1, while individuals in other categories are weighted in proportion to how likely they would have been in the base group relative to their actual group. These weights are then used to adjust the DID estimates.

Inverse-probability-weighted descriptive characteristics reported in Appendix Table H1 show that the differences in the baseline characteristics observed in the raw data largely disappeared.

Appendix Table H1. Descriptive characteristics by the policy intervention-control group and pre-post ACA period [inverse-probability-weighted].

	Non-TRICARE adults, Post-ACA	Non-TRICARE adults, Pre-ACA	TRICARE adults, Post-ACA	TRICARE adults, Pre-ACA
N	150,838 (24.9%)	150,619 (24.8%)	150,767 (24.8%)	154,603 (25.5%)
Race-ethnicity				
Non-Hispanic White	63,793 (42.3%)	64,119 (42.6%)	73,376 (48.7%)	70,403 (45.5%)
Non-Hispanic Black	27,895 (18.5%)	27,711 (18.4%)	25,511 (16.9%)	24,971 (16.2%)
Non-Hispanic All Other Race	15,062 (10.0%)	15,426 (10.2%)	17,381 (11.5%)	13,993 (9.1%)
Hispanic	44,088 (29.2%)	43,363 (28.8%)	34,499 (22.9%)	45,236 (29.3%)
Age [mean (SD)]	40.379 (13.423)	40.318 (13.228)	39.009 (15.265)	38.593 (15.528)
Sex				
Male	70,735 (46.9%)	70,886 (47.1%)	76,619 (50.8%)	80,164 (51.9%)
Female	80,103 (53.1%)	79,733 (52.9%)	74,148 (49.2%)	74,439 (48.1%)
Marital status				
Married	71,655 (47.5%)	71,830 (47.7%)	68,714 (45.6%)	70,379 (45.5%)
Widow	2,679 (1.8%)	2,613 (1.7%)	2,842 (1.9%)	3,210 (2.1%)
Divorced	16,698 (11.1%)	16,427 (10.9%)	13,703 (9.1%)	15,597 (10.1%)
Separated	4,685 (3.1%)	4,578 (3.0%)	4,460 (3.0%)	7,032 (4.5%)
Single	55,121 (36.5%)	55,170 (36.6%)	61,046 (40.5%)	58,385 (37.8%)
Census region				
South	56,516 (37.5%)	56,074 (37.2%)	52,622 (34.9%)	54,708 (35.4%)
Northeast	24,253 (16.1%)	24,534 (16.3%)	25,104 (16.7%)	27,567 (17.8%)
Midwest	29,371 (19.5%)	29,400 (19.5%)	35,359 (23.5%)	38,136 (24.7%)
West	40,698 (27.0%)	40,611 (27.0%)	37,681 (25.0%)	34,192 (22.1%)
Education				
< High school	33,818 (22.9%)	36,901 (24.5%)	28,626 (19.7%)	40,231 (26.0%)

High school	58,933 (39.8%)	58,503 (38.8%)	61,796 (42.6%)	58,473 (37.8%)
College	45,588 (30.8%)	45,713 (30.4%)	45,859 (31.6%)	48,677 (31.5%)
➢ College	9,583 (6.5%)	9,501 (6.3%)	8,684 (6.0%)	7,222 (4.7%)
Unemployment				
No	106,976 (70.9%)	107,168 (71.2%)	108,741 (72.1%)	110,413 (71.4%)
Yes	43,862 (29.1%)	43,451 (28.8%)	42,026 (27.9%)	44,190 (28.6%)
Family income				
Poor (≤100% FPL)	27,313 (18.1%)	26,891 (17.9%)	25,580 (17.0%)	25,369 (16.4%)
Near poor (100% to < 125% FPL)	8,135 (5.4%)	8,039 (5.3%)	6,943 (4.6%)	9,089 (5.9%)
Low (125% to < 200% FPL)	23,507 (15.6%)	23,362 (15.5%)	21,890 (14.5%)	22,964 (14.9%)
Middle (200% to < 400% FPL)	44,727 (29.7%)	44,659 (29.7%)	44,568 (29.6%)	47,542 (30.8%)
High (>= 400% FPL)	47,156 (31.3%)	47,667 (31.6%)	51,786 (34.3%)	49,640 (32.1%)

Provided below is the result from the multinomial propensity-score model.

Appendix Table H2. Multinomial propensity-score model (n=230,747)

	Outcome categories (Base: Non-TRICARE adults, Post-ACA)		
	Non-TRICARE adults, Pre-ACA	TRICARE adults, Post-ACA	TRICARE adults, Pre-ACA
Race/ethnicity (Ref: Non-Hispanic White)			
Black	-0.035**	0.305***	0.039
	(0.013)	(0.048)	(0.073)
Other Race	-0.161***	-0.288***	-0.237*
	(0.017)	(0.066)	(0.098)
Hispanic	-0.206***	-0.597***	-0.925***
	(0.012)	(0.057)	(0.090)
Age	-0.017***	-0.007**	-0.011**
	(0.001)	(0.002)	(0.004)
Female	0.012	-0.153	0.673***
	(0.029)	(0.129)	(0.197)
AgeX Female	-0.001	-0.005	-0.019***
	(0.001)	(0.003)	(0.004)
Marital status (Ref: Married)			
Widow	-0.053	0.486***	-0.030
	(0.035)	(0.105)	(0.189)
Divorced	-0.120***	-0.402***	-0.560***
	(0.015)	(0.064)	(0.098)
Separated	-0.219***	-0.205	-0.024
	(0.027)	(0.115)	(0.150)
Single	-0.410***	-1.127***	-1.333***
	(0.012)	(0.057)	(0.090)
Census region (Ref: East)			
Northeast	-0.009	-1.293***	-1.436***
	(0.014)	(0.074)	(0.116)
Midwest	0.003	-0.895***	-1.284***
	(0.013)	(0.057)	(0.096)
West	0.071***	-0.324***	-0.343***
	(0.012)	(0.046)	(0.068)
Education (Ref: < High school)			
High school	-0.045***	0.709***	0.712***
	(0.012)	(0.064)	(0.092)
College	-0.515***	0.838***	0.367***
	(0.014)	(0.067)	(0.099)
> college	-0.126***	0.802***	0.322*
	(0.021)	(0.088)	(0.137)
Unemployed	-0.088***	0.889***	0.725***
	(0.011)	(0.042)	(0.063)
Family income (Ref: <100%FPL)			
Near poor (100% to < 125% FPL)	0.032	0.395***	0.276
	(0.022)	(0.112)	(0.179)

Low (125% to < 200% FPL)	0.054***	0.558***	0.591***
	(0.016)	(0.082)	(0.125)
Middle (200% to < 400% FPL)	0.095***	0.827***	0.964***
	(0.015)	(0.073)	(0.111)
High (>= 400% FPL)	0.075***	1.092***	1.127***
	(0.016)	(0.075)	(0.115)
Having a chronic condition	0.591***	0.175***	0.941***
	(0.010)	(0.042)	(0.060)
Intercept	0.153***	-4.363***	-5.056***
	(0.030)	(0.134)	(0.207)

Note: Standard errors are in parentheses.
*** p<.001, ** p<.01, * p<.05
